# The optimal time of B-type natriuretic peptide sampling associated with post-myocardial infarction remodelling after primary percutaneous coronary intervention

**DOI:** 10.5830/CVJA-2013-024

**Published:** 2013-06

**Authors:** Hyunmin Choi, Hee-Jeong Yoon, Joon-Hyung Doh, Byung-Su Yoo, Min-Soo Ahn, Jang-Young Kim, Seung-Hwan Lee, Junghan Yoon

**Affiliations:** Top Care Cardiovascular Centre, Gumdan Top Hospital, Dangha-dong, Seo-gu, Incheon, South Korea; Top Care Cardiovascular Centre, Gumdan Top Hospital, Dangha-dong, Seo-gu, Incheon, South Korea; Division of Cardiology, Ilsan Paik Hospital, Inje University College of Medicine, Goyang, Republic of Korea; Division of Cardiology, Wonju Christian Hospital, Wonju College of Medicine, Yonsei University, Wonju, Republic of Korea; Institute of Lifelong Health, Wonju Christian Hospital, Wonju College of Medicine, Yonsei University, Wonju, Republic of Korea; Division of Cardiology, Wonju Christian Hospital, Wonju College of Medicine, Yonsei University, Wonju, Republic of Korea; Institute of Lifelong Health, Wonju Christian Hospital, Wonju College of Medicine, Yonsei University, Wonju, Republic of Korea; Division of Cardiology, Wonju Christian Hospital, Wonju College of Medicine, Yonsei University, Wonju, Republic of Korea; Institute of Lifelong Health, Wonju Christian Hospital, Wonju College of Medicine, Yonsei University, Wonju, Republic of Korea; Division of Cardiology, Wonju Christian Hospital, Wonju College of Medicine, Yonsei University, Wonju, Republic of Korea; Institute of Lifelong Health, Wonju Christian Hospital, Wonju College of Medicine, Yonsei University, Wonju, Republic of Korea; Division of Cardiology, Wonju Christian Hospital, Wonju College of Medicine, Yonsei University, Wonju, Republic of Korea; Institute of Lifelong Health, Wonju Christian Hospital, Wonju College of Medicine, Yonsei University, Wonju, Republic of Korea

**Keywords:** B-type natriuretic peptide, remodelling, myocardial infarction

## Abstract

**Aims:**

To find the optimal time to evaluate plasma B-type natriuretic peptide (BNP), which is related to post-myocardial infarction remodelling (PMIR), we measured serial plasma BNP levels according to time protocols after primary percutaneous coronary intervention (PCI).

**Background:**

It has been established that plasma BNP levels can predict the development of PMIR in patients with ST-elevation myocardial infarction (STEMI). However, the time of plasma BNP sampling associated with PMIR is still controversial.

**Methods:**

We analysed 42 patients who were diagnosed as PMIR on six-month follow-up echocardiography among 131 patients with STEMI. We then compared clinical variables including plasma BNP between the remodelling group and the non-remodelling group. The plasma BNP level was obtained on hospital admission (acute phase), at two to five days (early phase), three to four weeks (late phase) and at the six-month follow up (long term).

**Results:**

Early-phase and long-term BNP levels were higher in the remodelling group. The serial plasma BNP levels, according to study protocols, showed a biphasic pattern of elevation. In multiple logistic regression analyses, early-phase BNP [odds ratio (OR): 1.013, *p* < 0.01] and acute-phase BNP levels (OR: 1.007, *p* = 0.02) were independent predictors of PMIR. However, early-phase BNP level was statistically a more powerful predictor of PMIR during follow up.

**Conclusion:**

Consecutive BNP levels after primary PCI showed a biphasic peak elevation during follow up. Earlyphase plasma BNP level was an independent predictor of PMIR in patients with STEMI.

## Abstract

Post-myocardial infarction remodelling (PMIR) in patients with ST-elevation myocardial infarction (STEMI) is detrimental to normal left ventricular (LV) systolic function and is associated with heart failure and death due to cardiovascular events.^1^ Although an early reperfusion strategy such as primary percutaneous coronary intervention (PCI) has become widespread in recent years, a significant percentage of STEMI patients still suffer from PMIR.

Anterior wall infarction, peak levels of creatine kinase myocardial band (CK-MB) and troponin I, LV systolic dysfunction, and wall motion score index (WMSI) have been associated with the development of PMIR.^2^ Also, increased expression of B-type natriuretic peptide (BNP) has been suggested to be an indicator of PMIR, so plasma BNP levels can be used as diagnostic and monitoring tools for PMIR in patients with STEMI.^3^,^4^

Plasma BNP levels in patients with STEMI have shown a positive correlation with the degree of LV systolic and diastolic dysfunction.^5^ Although the prognostic significance of BNP levels has been elucidated, a suitable point in time for BNP sampling related to PMIR has not been established. Several studies have reported that plasma BNP levels measured at hospital admission or in the acute phase were meaningful predictors of PMIR,^5^,^6^ whereas others have stated that later sampling during follow up was associated with PMIR.^7^,^8^

We evaluated serial changes in plasma BNP levels after successful revascularisation using primary PCI. We identified a suitable point in time for BNP sampling as an independent predictor of PMIR in patients with STEMI.

## Methods

The research protocol was approved by the Committee on Ethics and Research of Wonju Christian Hospital (Wonju College of Medicine, Yonsei University, Wonju, Republic of Korea). Written informed consent was obtained from each patient.

One hundred and thirty-one STEMI patients were the study subjects. All patients were admitted to Wonju Christian Hospital. They had received reperfusion therapy using primary PCI within 12 hours of symptom onset and had blood sampling with a planned schedule for BNP. We then compared clinical variables including plasma BNP levels and echocardiographic data between the remodelling (RG) and non-remodelling groups (NRG). The study period was from April 2006 to March 2009.

Inclusion criteria were ischaemic chest pain lasting ≥ 30 min; electrocardiographic ST-segment elevation > 0.1 mV in two or more limb leads, or > 2 mV in two or more precordial leads or new-onset left bundle branch block; and elevation in the level of CK-MB or troponin I to ≥ twice the normal range.

Exclusion criteria were previous myocardial infarction (MI); severe valvular heart disease; cardiomyopathy; impaired renal function (creatinine > 1.5 mg/dl); inadequate quality of echocardiographic images; cardiogenic shock (initial systolic blood pressure < 90 mmHg); advanced heart failure (Killip class ≥ III); or life-limiting non-cardiac disease.

Success of revascularisation using primary PCI was defined as residual stenosis < 50% and if coronary flow in the culprit vessel after primary PCI resulted in thrombolysis in myocardial infarction (TIMI) grade ≥ 2. The primary PCI procedure and type of stent used were at the discretion of the interventional cardiologist.

Coronary angiographic analysis during primary PCI was performed by interventional cardiologists in the Ilsan Paik Hospital cardiovascular centre, who were blinded to clinical and plasma BNP findings. All patients were prescribed aspirin (300 mg p.o.), clopidogrel (600 mg p.o.), and heparin (70 IU/kg p.o.) before the procedure. Each patient was maintained on aspirin (100 mg) and clopidogrel (75 mg) for ≥ 12 months after revascularisation. Also, most patients received β-blockers, calcium-channel blockers, angiotensin receptor blockers (ARBs) or angiotensin-converting enzyme inhibitors (ACEIs), and statins at the discretion of the attending physician.

Blood samples were taken for BNP measurement on hospital admission (acute phase), at two to five days (early phase), three to four weeks (late phase), and at six months (long term) after symptom onset. All plasma samples were obtained in plastic tubes containing potassium ethylene diamine tetra-acetic acid (EDTA; Becton Dickinson, Franklin Lakes, NJ, USA) with amounts that ranged from 3–5 ml. All samples were centrifuged, and plasma was tested singly for BNP using the Biosite Triage Assay, a point-of-care device that uses a fluorescence immunoassay technique (Biosite, San Diego, CA, USA).

The total coefficient of variation at different levels of plasma BNP was reported to be < 7% using control samples provided by the manufacturer. The sensitivity for BNP in these measurements ranges from 5–5 000 pg/ml. Levels of CK-MB and troponin I were evaluated after symptom onset. The peak release of CK-MB and troponin I was determined every four hours after hospital admission for three days.

Two-dimensional echocardiography was undertaken at baseline and at the six-month follow up. Echocardiographic examinations and data were obtained using a commercially available imaging system (Vivid 7; GE Medical Systems, Milwaukee, WI, USA). Echocardiograhic data were sent to the echocardiography laboratory in Ilsan Paik Hospital cardiovascular centre and analysed by echocardiography physicians blinded to the laboratory data.

Apical four- and two-chamber views as well as apical longaxis views were obtained from all patients. To assess regional wall motion abnormalities, the wall of the LV was divided into 16 segments, as recommended by the American Society of Echocardiography.^9^ For each segment, the WMSI was derived. LV end-diastolic volume (LVEDV), LV end-systolic volume (LVESV) and LV ejection fraction (LVEF) were calculated using a modified version of Simpson’s method.

Assessment of diastolic function was carried out by measuring the mitral inflow pattern with pulsed-wave Doppler [E/A ratio, and deceleration time (DT) of the E wave], pulmonary venous inflow, and tissue Doppler velocities of the mitral annulus. The ratio of early diastolic mitral annulus velocity (E/E′) was used as an indicator for LV filling pressures.^10^

PMIR was defined as > 20% increment in LVEDV estimated at the six-month follow-up echocardiography compared with baseline results using a modified version of Simpson’s method.^2^ Intra- and inter-observer variability of LVEDV and LVESV was < 5% in this study.

## Statistical analyses

Data were analysed using the SPSS statistical package, version 15.0 (SPSS Incorporated, Chicago, IL, USA). Data are mean ± SD for continuous variables and frequency with percentages for categorical variables. Because mean BNP levels were uneven, natural log transformation was used in the regression analyses to satisfy modelling assumptions.

Continuous variables were compared using the paired Student’s *t*-test. Categorical variables were compared using chi-square analyses. Differences in proportions were compared using Pearson’s chi-square test. Repeated-measures analysis of variance (ANOVA) was used to analyse inter- and intra-group differences between the RG and NRG with regard to plasma BNP levels; *p* < 0.05 was considered significant. Univariate and multiple logistic regression analyses were carried out to estimate independent predictors of PMIR. Variable selection in multivariable modelling was based on statistical significance from univariate analysis.

The optimal time of BNP sampling for the prediction of PMIR was determined by a multivariate model. The BNP cut-off value for prediction of PMIR was assessed by receiver operator characteristic (ROC) curve analyses. The predictive value of plasma BNP level for PMIR was evaluated using estimation of the area under the curve (AUC) separately for each parameter.

## Results

The clinical characteristics of the study population are shown in Table 1. All patients treated with primary PCI received at least one stent implantation. PMIR was detected in 42 patients. The mean age was older in the RG (RG vs NRG; 63.1 ± 11.9 vs 58.1 ± 11.1 years, *p* = 0.02). The mean time from symptom onset to reperfusion was later in the RG, but was not statistically significant (RG vs NRG; 5.4 ± 2.3 vs 4.8 ± 2.2 h, *p* = 0.07).

**Table 1.** Baseline Clinical Characteristics Between Non-Remodelling And Remodelling Groups

*Variable*	*Non-remodelling group (n = 89)*	*Remodelling group (n = 42)*	*p*
Age (years)	58.1 ± 11.1	63.1 ± 11.9	0.02
Males, *n* (%)	68 (76.4)	26 (61.9)	0.14
Diabetes mellitus, *n* (%)	26 (29.2)	10 (23.8)	0.68
Hypertension,* *n* (%)	46 (51.7)	18 (42.9)	0.35
Current smoker, *n* (%)	49 (55.1)	23 (54.8)	0.47
Hypercholesterolaemia,^†^ *n* (%)	49 (55.1)	22 (52.4)	0.45
Time from symptom onset to to reperfusion (h)	4.8 ± 2.1	5.4 ± 2.3	0.07
Killip class I, *n* (%)	41 (44.9)	17 (40.5)	0.26
NYHA class I, *n* (%)	70 (78.7)	24 (57.1)	0.03
Peak CK-MB (ng/ml)	170.9 ± 109.9	246.8 ± 88.1	< 0.01
Peak troponin I (ng/ml)	33.7 ± 25.1	48.3 ± 28.3	< 0.01
Discharge medications			
Aspirin, *n* (%)	89 (100)	42 (100)	
Clopidogrel, *n* (%)	89 (100)	42 (100)	
β-blockers, *n* (%)	81 (91.1)	36 (85.7)	0.22
ACEIs or ARBs, *n* (%)	85 (95.5)	38 (90.5)	0.49
Diuretics, *n* (%)	44 (49.4)	22 (52.4)	0.41
Statins, *n* (%)	86 (96.6)	40(97.6)	0.86

Data are mean ± SD or numbers (percentage). *Systolic pressure > 140 mmHg and/or diastolic pressure > 90 mmHg or receiving antihypertensive drugs. ^†^Total cholesterol > 220 mg/dl and/or low-density lipoprotein cholesterol > 130 mg/dl or receiving statin therapy. NYHA, New York Heart Association; CK-MB, creatinine kinase myocardial band; ACEI, angiotensin-converting enzyme inhibitor; ARB, angiotensin II receptor blocker.

There were significant differences in the percentage of New York Heart Association class I between the two groups (RG vs NRG 57.1 vs 78.7%, *p* = 0.03). Moreover, mean peak levels of CK-MB (RG vs NRG; 246.8 ± 88.1 vs 170.9 ± 109.9 ng/ml, *p* < 0.01) and troponin I (RG vs NRG; 48.3 ± 28.3 vs 33.7 ± 25.1 ng/ml, *p* < 0.01) were significantly higher in the RG. At hospital discharge, all patients received aspirin and clopidogrel, and there was no statistical difference in percentage use of β-blockers, ACEIs, ARBs, diuretics and statins between the two groups.

The baseline angiographic and procedural characteristics of the study population are listed in Table 2. With regard to the extent of coronary artery disease (CAD), the proportion of multi-vessel disease was similar between the two groups [RG vs NRG; 41.6% (*n* = 17) vs 42.9% (*n* = 37), *p* = 0.79]. In the RG, the most frequently involved coronary artery was the left anterior descending artery [RG vs NRG; 61.9% (*n* = 26) vs 42.7% (*n* = 38), *p* = 0.04].

**Table 2.** Baseline Procedural Characteristics Between Non-Remodelling And Remodelling Groups

*Variable*	*Non-remodelling group (n = 89)*	*Remodelling group (n = 42)*	*p*
Multi-vessel disease, *n* (%)	37 (41.6)	18 (42.9)	0.79
IRA			
LAD, *n* (%)	38 (42.7)	26 (61.9)	0.04
LCX, *n* (%)	7 (7.9)	5 (11.9)	0.40
RCA, *n* (%)	44 (49.4)	11 (26.2)	0.01
Stent type (%)			
DES, *n* (%)	81 (91.1)	38 (90.5)	0.72
BMS, *n* (%)	8 (8.9)	4 (9.5)	0.69
DES type (%)			
SES, *n* (%)	29 (35.8)	16 (42.1)	0.43
PES, *n* (%)	39 (48.1)	18 (47.4)	0.47
ZES, *n* (%)	13 (16.1)	4 (10.5)	0.25
TIMI grade 3 after PCI, *n* (%)	84 (94.4)	36 (85.7)	0.09
Number of stents	1.25 ± 0.53	1.27 ± 0.36	0.33

Data are mean ± SD or numbers (percentage). IRA, infarct-related artery; LAD, left anterior descending artery; LCX, left circumflex artery; RCA, right coronary artery; BMS, bare-metal stent; DES, drug-eluting stent; SES, sirolimus-eluting stent; PES, paclitaxel-eluting stent; ZES, zotarolimus-eluting stent; TIMI, thrombolysis in myocardial infarction; PCI, percutaneous coronary intervention.

Almost 90% of patients who underwent primary PCI received drug-eluting stent (DES) implantation. No difference was observed in terms of the proportion of DES or bare-metal stent (BMS) implantation between the two groups. Compared with a zotarolimus-eluting stent (ZES), we mainly used a sirolimuseluting stent (SES) and paclitaxel-eluting stent (PES).

In addition, the final thrombolysis in myocardial infarction (TIMI) grade 3 flow after primary PCI [RG vs NRG; 85.7% (*n* = 36) vs 94.4% (*n* = 84), *p* = 0.09] and the number of stents per patient (RG vs NRG; 1.25 ± 0.53 vs 1.27 ± 0.36, *p* = 0.33) showed no significant differences between the two groups. We seldom used 2b/3a glycoprotein inhibitors during primary PCI [RG vs NRG; 9.5% (*n* = 4) vs 7.9% (*n* = 7), *p* = 0.78].

Baseline and follow-up haemodynamic parameters as well as diastolic dysfunction of the study population are listed in Table 3. Mean baseline LVESV and E/E′ were significantly higher in the RG than in the NRG. Also, mean baseline LVEF was significantly reduced in the RG. Follow-up echocardiography was performed at a mean of 6.5 ± 1.1 months after primary PCI.

**Table 3.** Baseline And Follow-Up Echocardiographic Characteristics Between Non-Remodelling And Remodelling Groups

*Variable*	*Non-remodelling group (n = 89)*	*Remodelling group (n = 42)*	*p*
Haemodynamics			
Baseline			
LVESV (ml)	31.6 ± 16.8	37.2 ± 19.2	0.03
LVEDV (ml)	71.1 ± 20.2	74.4 ± 16.1	0.36
LVEF (%)	57.0 ± 9.5	50.3 ± 9.3	0.00
DT (ms)	211.7 ± 42.7	203.9 ± 46.1	0.10
E/E′	11.0 ± 4.3	14.5 ± 2.8	< 0.01
Follow up			
LVESV (ml)	28.9 ± 14.2	49.3 ± 17.3	< 0.01
LVEDV (ml)	69.9 ± 19.3	94.7 ± 21.4	< 0.01
LVEF (%)	60.2 ± 10.6	48.9 ± 10.7	< 0.01
DT (ms)	236.7 ± 42.0	204.7 ± 49.3	< 0.01
E/E′	7.9 ± 3.8	11.2 ± 6.2	< 0.01
Diastolic dysfunction (%)			
Baseline			
Grade 1 (%)	56 (62.9)	21 (50.0)	0.16
Grade 2 (%)	22 (24.7)	17 (40.5)	0.07
Grade 3 (%)	0 (0)	2 (4.8)	0.04
Follow up			
Grade 1 (%)	76 (85.4)	26 (61.9)	< 0.01
Grade 2 (%)	3 (3.4)	9 (21.4)	< 0.01
Grade 3 (%)	0 (0)	5 (11.9)	< 0.01

Data are mean ± SD or numbers (percentage). LV, left ventricular; ESV, end-systolic volume; EDV, end-diastolic volume; EF, ejection fraction; DT, deceleration time.

At the six-month follow up, LVESV and LVEDV in the RG were increasing compared with baseline values. Mean six-month follow-up LVEF did not show notable changes compared with baseline. Although baseline diastolic dysfunction was not significantly different between the two groups, six-month follow-up diastolic dysfunction in the NRG showed a notable improvement. Mean six-month follow-up E/E′ in the RG was decreased in both groups, but mean E/E′ was significantly higher in the RG (RG vs NRG; 11.2 ± 6.2 vs 7.9 ± 3.8, *p* < 0.01).

**Fig. 1.**
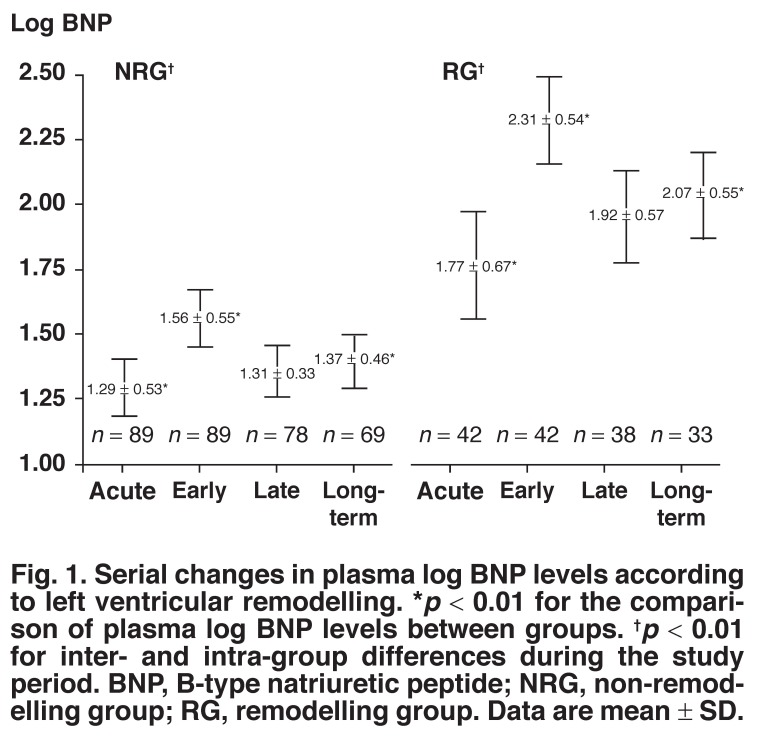
Serial changes in plasma log BNP levels according to left ventricular remodelling. *p < 0.01 for the comparison of plasma log BNP levels between groups. ^†^*p* < 0.01 for inter- and intra-group differences during the study period. BNP, B-type natriuretic peptide; NRG, non-remodelling group; RG, remodelling group. Data are mean ± SD.

Mean time of plasma BNP measurements in the early phase was 2.8 ± 0.5 days, in the late phase 3.7 ± 0.6 weeks, and longterm 6.3 ± 0.6 months after symptom onset. In the RG, mean plasma log BNP levels were significantly elevated in the acute (RG vs NRG; 1.77 ± 0.67 vs 1.29 ± 0.53, *p* < 0.01) and early phase (RG vs NRG; 2.31 ± 0.54 vs 1.56 ± 0.55, *p* < 0.01), and long term (RG vs NRG; 2.07 ± 0.55 vs 1.37 ± 0.46, *p* < 0.01) (Fig. 1).

Mean plasma BNP levels were significantly different between the two groups (*p* < 0.01, repeated measures ANOVA) and during each time phase (*p* < 0.01, repeated measures ANOVA). Compared with the NRG, the RG mean plasma BNP levels were prominently elevated in the early phase and long term. This biphasic peak elevation of plasma BNP level was a characteristic feature of the RG. As we examined plasma BNP measurements throughout each study period, plasma BNP levels in the RG were consistently higher than in the NRG.

Univariate regression analyses were conducted to determine a surrogate marker for PMIR independent of other predictors. The age, time from symptom to reperfusion, peak levels of troponin I and CK-MB, LVEF and E/E′ were significantly associated with PMIR at the six-month follow up.

Hierarchical multiple regression analyses for optimal time of PMIR were constructed (Table 4). In the multivariate model, after adjusting for age, gender, time from symptom to reperfusion, troponin I level, CK-MB level, LVEF, E/E′ and WMSI, acuteand early-phase BNP were identified as independent predictors of PMIR at the six-month follow up.

**Table 4.** Multiple Logistic Regression Analysis To Evaluate The Time Point Of Plasma BNP Sampling That Is Closely Associated With LV Remodelling. Stepwise Adjustment Of Different Factors Including Age, Gender, Time From Symptom Onset To Reperfusion, CK-MB Level, Troponin-I LEVEL, E/E′ And WMSI

*Variable*	*Odds ratio*	*95% Confidence interval*	*p*
Model 1			
Age, gender adjusted			
Acute BNP	1.006	1.001–1.011	0.02
Early BNP	1.011	1.007–1.016	< 0.01
Late BNP	1.005	0.998–1.011	0.15
Long-term BNP	1.010	1.004–1.016	1.004–1.016 0.01
Model 2			
Time from symptom onset to reperfusion adjustment			
Acute BNP	1.005	0.999–1.010	0.08
Early BNP	1.011	1.006–1.016	< 0.01
Late BNP	1.004	0.997–1.010	0.24
Long-term BNP	1.009	1.002–1.015	0.01
Model 3			
Tn-I, CK-MB adjusted			
Acute BNP	1.008	1.002–1.014	0.01
Early BNP	1.012	1.006–1.018	< 0.01
Late BNP	1.004	0.997–1.012	0.22
Long-term BNP	1.007	1.001–1.014	0.03
Model 4			
LVEF-, E/E′-, WMSI-adjusted			
Acute BNP	1.007	1.001–1.014	0.02
Early BNP	1.013	1.006–1.019	< 0.01
Late BNP	1.004	0.996–1.012	0.36
Long-term BNP	1.005	0.998–1.012	0.13

*p-values are based on the multiple regression analysis. BNP, B-type natriuretic peptide; Tn-I, troponin I; CK-MB, creatinine kinase myocardial band; LVEF, left ventricular ejection fraction.

**Fig. 2.**
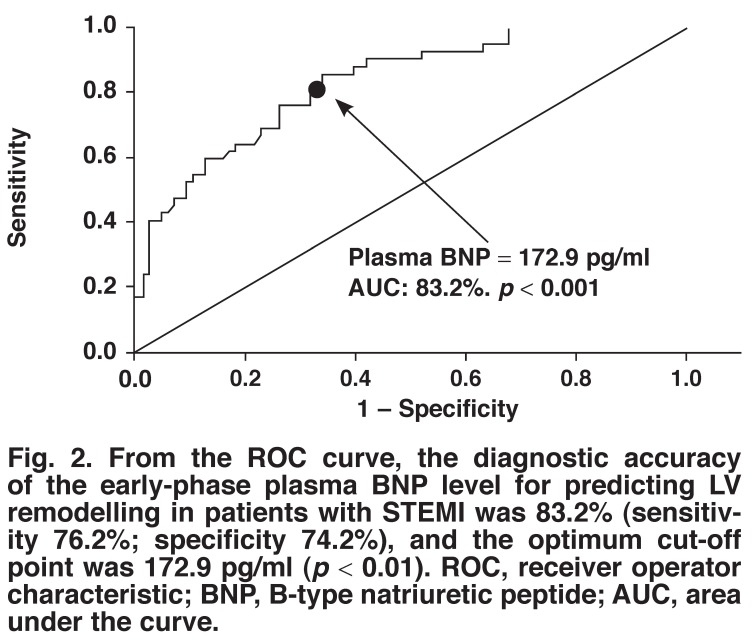
From the ROC curve, the diagnostic accuracy of the early-phase plasma BNP level for predicting LV remodelling in patients with STEMI was 83.2% (sensitivity 76.2%; specificity 74.2%), and the optimum cut-off point was 172.9 pg/ml (*p* < 0.01). ROC, receiver operator characteristic; BNP, B-type natriuretic peptide; AUC, area under the curve.

Among the time phases, early-phase BNP was a meaningful predictor of LV remodelling. ROC curves for early-phase plasma BNP levels for the prediction of PMIR are shown in Fig. 2. The AUC of early-phase BNP levels for predicting PMIR was 0.83 with a cut-off value of 172.9 pg/ml. Early-phase plasma BNP levels showed a sensitivity of 76.2% and a specificity of 74.2%.

## Discussion

The main finding of the present study was that the appropriate plasma BNP sampling time that reflected the development of PMIR in patients with STEMI was the early phase, from two to five days after the onset of STEMI. Several studies focusing on the association between plasma BNP and PMIR have confirmed that elevated BNP is a marker for LV systolic dysfunction and a significant prognostic marker for morbidity and mortality in patients with STEMI.^11^,^12^ However, some discrepancies exist in the optimal time point of plasma BNP sampling that is associated with the prediction of PMIR.

Some studies reported that plasma BNP levels at one to four days were strongly related to PMIR after STEMI.^13^-^15^ Other studies suggested that plasma BNP levels on hospital admission were associated with PMIR at the six-month follow up in patients with STEMI.^5^,^16^ One study demonstrated that BNP sampling three to four weeks after the onset of STEMI was significantly correlated with PMIR.^7^

The results of the present study conflict with several studies reporting that BNP sampling three to four weeks after the onset of STEMI was significantly correlated with PMIR. Although our results showed concordance between plasma BNP levels at hospital admission and PMIR, plasma BNP levels measured from two to five days represented a more powerful predictor of PMIR in patients with STEMI. These discrepancies may have been due to an inhomogeneous study population, varying time from symptom onset to reperfusion, reperfusion strategy, infarct-related arteries, underlying medical conditions, and timing of BNP measurement.

Plasma BNP level was increased in the acute and early phase and decreased during follow up in the present study. Plasma BNP may be synthesised in the ventricular myocardium and released into the bloodstream in response to multiple stimuli, including ischaemia, inflammation, ventricular volume overload, pressure overload, and reperfusion injury.^17^,^18^ Myocardial ischaemia, the inflammatory response, ventricular volume overload or pressure overload before reperfusion may occur simultaneously in patients with STEMI. Importantly, myocardial ischaemia and inflammatory stimuli can be aggravated by reperfusion during primary PCI.^19^

Microcirculatory obstruction may occur via distal embolisation, and infarct expansion during PCI may affect the elevation of plasma BNP levels.^20^ Post-PCI plasma BNP level was consistently higher than pre-PCI plasma BNP level in patients with STEMI, and those who showed elevated post-PCI plasma BNP levels were expected to undergo PMIR or cardiac death.^21^

Accumulation of intracellular calcium in infarcted myocardium before reperfusion does not stimulate or increase the synthesis and secretion of BNP in the plasma.^22^ Even if plasma BNP level at hospital admission could be a sufficient surrogate marker for PMIR, it cannot reflect a broad spectrum of myocardial damage, including reperfusion injury.

Plasma BNP levels after reperfusion in STEMI appeared to be higher in our study than in several studies on patients with acute or chronic heart failure.^15^,^23^ Hence, myocardial ischaemia may be a stronger factor than ventricular volume overload or pressure overload as a stimulus for BNP secretion. Therefore, ventricular volume overload or pressure overload before reperfusion may be non-specific in Killip class I–II or in haemodynamically stable patients with STEMI.

Plasma BNP level measured from two to five days after reperfusion correlated not only with ischaemic injury but also reperfusion injury. This may be important for using post-PCI plasma BNP level as an integrated biomarker of total myocardial damage. Also, we demonstrated that plasma BNP level could be a useful and significant predictor of PMIR that was not inferior or superior to other established predictors, including age, peak level of CK-MB and troponin I reflecting infarct size, as well as echocardiographic LVEF and diastolic filling parameters.

Studies in STEMI and non-STEMI patients have demonstrated that a biphasic pattern of plasma BNP levels reflects the major damage to the myocardium and subsequent LV systolic dysfunction.^4^,^24^ Peak plasma BNP elevations in the present study were observed in the early phase and long term during the follow-up periods. Compared with the NRG, plasma BNP levels were more prominent in the early and long-term phases, so the pattern of plasma BNP elevation was similar to that observed in the previous study. Although BNP level three to four weeks after the onset of STEMI reflecting the second peak of plasma BNP has been reported,^7^ plasma BNP level at six months after the onset of STEMI appeared to represent the second peak in the current study.

It has been reported that Doppler-derived E/E′,^25^ and DT^26^ are relevant measurements of elevated LV filling pressure and LV dilation in patients with STEMI. We also evaluated and analysed the diastolic parameters of echocardiography for the prediction of PMIR. Among the parameters, initial Doppler-derived E/E′ was significantly correlated with PMIR in a multiple regression model. Although studies have suggested that Doppler-derived DT is closely related to the risk of PMIR after STEMI, DT cannot reflect elevated mean LV diastolic pressure in patients with preserved systolic function.^25^,^26^ In the present study, plasma BNP levels at two to five days and initial Doppler-derived E/E′ proved to be significant predictors of PMIR.

We focused on the background of post-PCI plasma BNP elevation after successful coronary reperfusion. Although primary PCI is the optimal therapy in patients with STEMI, PMIR is a complication that may confound the prognosis. Post-PCI plasma BNP levels could be affected by myocardial ischaemia and inflammatory activation that follows reperfusion. Therefore, we should create an active management plan that attenuates myocardial ischaemia and inflammatory activation before and after primary PCI.

The present study had two main limitations. First, this study had a small sample size of PMIR patients. Second, a distribution of the infract-related artery and time from symptom onset to reperfusion was uneven because the clinical situation of each patient was different.

## Conclusion

Elevated plasma BNP level was an independent predictor for PMIR. The optimal timing of plasma BNP measurement was in the early phase after the onset of STEMI. We should continue to use plasma BNP level to define its potential role in monitoring for PMIR.
